# Rapidly Progressive Pulmonary Alveolar Proteinosis Following Cryptococcal Infection: Case Report and Literature Review

**DOI:** 10.1002/rcr2.70114

**Published:** 2025-02-10

**Authors:** Samuel Cartmel Brookes, Alexandra Sadler, Alexander Troelnikov, Pravin Hissaria, Michael Vickery Brown, Phan Nguyen, Julia Kim, Paroma Sarkar

**Affiliations:** ^1^ Department of Thoracic Medicine Royal Adelaide Hospital Adelaide South Australia Australia; ^2^ Faculty of Health and Medical Sciences University of Adelaide Adelaide South Australia Australia; ^3^ Immunology SA Pathology Adelaide South Australia Australia; ^4^ Immunology Department Royal Adelaide Hospital Adelaide South Australia Australia; ^5^ College of Medicine and Public Health Flinders University Adelaide South Australia Australia

**Keywords:** cryptococcosis, *Cryptococcus gattii*, extracorporeal membrane oxygenation, pulmonary alveolar proteinosis, whole lung lavage

## Abstract

Pulmonary alveolar proteinosis (PAP) is a rare disease caused by accumulation of sediment within alveoli. Cryptococcosis a fungal infection typically presenting with central nervous system (CNS) and pulmonary disease. Granulocyte‐macrophage colony‐stimulating factor antibodies are associated with PAP and elevated risk of cryptococcosis. The usual interval from cryptococcal infection to the onset of PAP spans several years. Here, we describe a case of a 24‐year‐old Aboriginal Australian woman with no prior medical history, who presented with seizures from CNS cryptococcosis, and subsequently developed rapidly progressive hypoxic respiratory failure secondary to autoimmune‐PAP within weeks of initial presentation. The rate and degree of respiratory failure necessitated urgent bilateral whole lung lavage (WLL) whilst on venovenous‐extracorporeal membrane oxygenation. Our report hopes to increase recognition of PAP in the Australian population, document the utility and risks of bilateral WLL in the critically unwell patient and provide an updated literature review of PAP and cryptococcal infection.

## Graphical abstract

Here, we present a rare case of rapidly progressive pulmonary alveolar proteinosis caused by GM‐CSF antibodies following cryptococcal CNS and pulmonary infection. Due to the rapidity, bilateral whole lung lavage on VV‐ECMO was required to achieve remission.

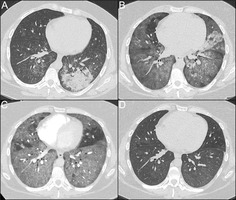

## 1 Introduction

Pulmonary alveolar proteinosis (PAP) is a rare cause of lung disease characterised by periodic acid‐Schiff (PAS) positive sediment accumulation within the alveolar space, impairing pulmonary gas exchange. Three aetiologies of PAP are recognised: congenital, secondary and autoimmune. Autoimmune‐PAP accounts for approximately 90% of cases and characterised by antibodies that cause dysfunction of alveolar macrophages. Granulocyte‐macrophage colony‐stimulating factor (GM‐CSF) is produced in the lung by type‐II pneumocytes, which activate macrophages to clear surfactant through GM‐CSF receptor binding leading to altered gene expression mediated by the JAK2/STAT5 pathway [1]. GM‐CSF antibodies interrupt this pathway leading to accumulation of alveolar sediment and development of PAP [1]. *Cryptococcus gattii* is a fungal organism that can cause severe infection, typically presenting with central nervous system or pulmonary disease in immunocompetent patients. It is reported that those with GM‐CSF antibodies are at elevated risk of cryptococcosis independent of PAP [2]. Typically, infections such as cryptococcosis precede diagnosis of PAP with case reports indicating a lag of multiple years [1, 3, 4, 5, 6, 7, 8, 9]. Here, we present the unusual case of a 24‐year‐old immunocompetent Aboriginal Australian woman with severe PAP occurring within 6‐weeks following cryptococcal infection. This close temporal association exacerbated diagnostic uncertainty which often surrounds PAP. The rapid progression to severe hypoxic respiratory failure necessitated veno‐venous extracorporeal membrane oxygenation (VV‐ECMO) to facilitate bilateral whole lung lavage (WLL). A review of the literature was conducted demonstrating the association of PAP with cryptococcosis and highlights the learning points of the complexity of diagnosis and importance of a collaborative, multi‐disciplinary approach to management.

## 2 Case Report

**FIGURE 1:**
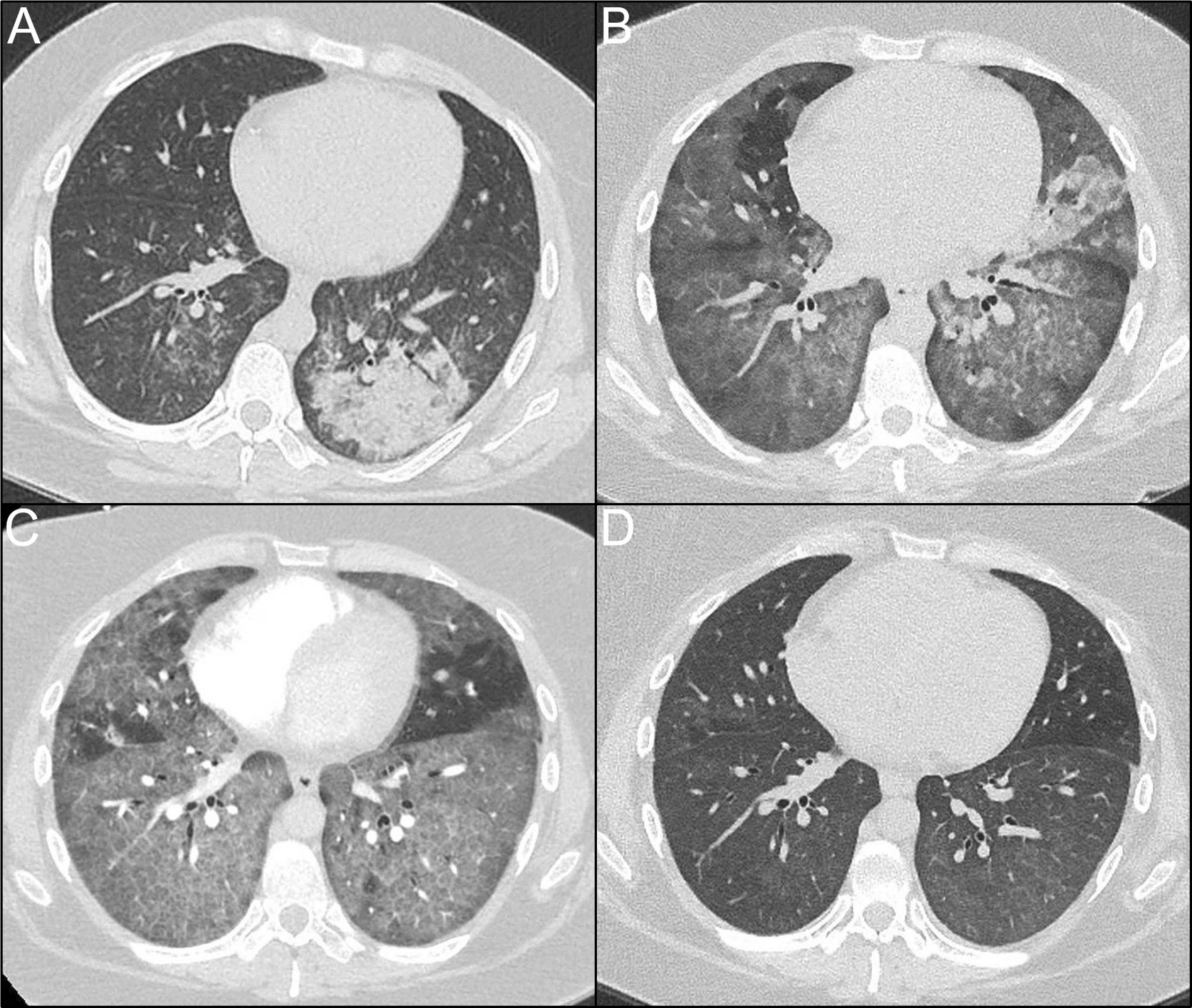
CT chest axial slices from initial presentation through to definitive management with whole lung lavage. (A) initial presentation with cryptococcal pneumonia with predominant left lower lobe consolidative change. (B) two weeks after initial presentation with progressive bilateral ground glass changes and early interlobular septal thickening which correlated with high CMV titre in BAL fluid. (C) Six weeks after initial presentation with progressive, confluent bilateral ground glass change and interlobular septal thickening in keeping with crazy paving pattern. (D) Four days post‐whole lung lavage with marked radiographic improvement.

A 24‐year‐old Aboriginal Australian woman presented after a tonic–clonic seizure. Clinical features of intracranial hypertension were identified and a microbiological diagnosis of *C. gattii* meningitis was made by cerebrospinal fluid (CSF) culture and elevated serum and CSF cryptococcal antigen titres. The patient had no significant medical history and unlimited exercise tolerance. She was a regular tobacco smoker of 10 cigarettes per day totalling 5 pack‐year duration, consumed up to 5 cannabis bongs per day and had occupational exposure to Australian flora whilst working as a manual labourer. Computed tomography (CT) chest identified asymptomatic left lower lobe consolidation (Figure 1A). Treatment consisted of corticosteroids, liposomal amphotericin‐B and 5‐flucytosine and complicated by immune reconstitution inflammatory syndrome (IRIS) and drug‐induced kidney injury. She was discharged with fluconazole, prednisolone and pneumocystis prophylaxis for a planned 6‐to‐12‐month duration.

Re‐admission occurred 2‐weeks later with symptoms of fever, cough and hypoxaemia, requiring low‐flow oxygen. CT chest demonstrated progressive consolidation in the upper lobes and widespread ground glass change (Figure 1B). Bronchoscopy yielded turbid fluid with high CMV titre (21,217 copies/mL). Microscopy, culture and sensitivity yielded growth of *Aspergillus nidulans* and *Staphylococcus epidermidis* . Both organisms were deemed contaminants. Treatment with intravenous ganciclovir and broad‐spectrum antibiotics were commenced and the patient discharged with oral fluconazole, valganciclovir and tapering prednisolone.

**FIGURE 2:**
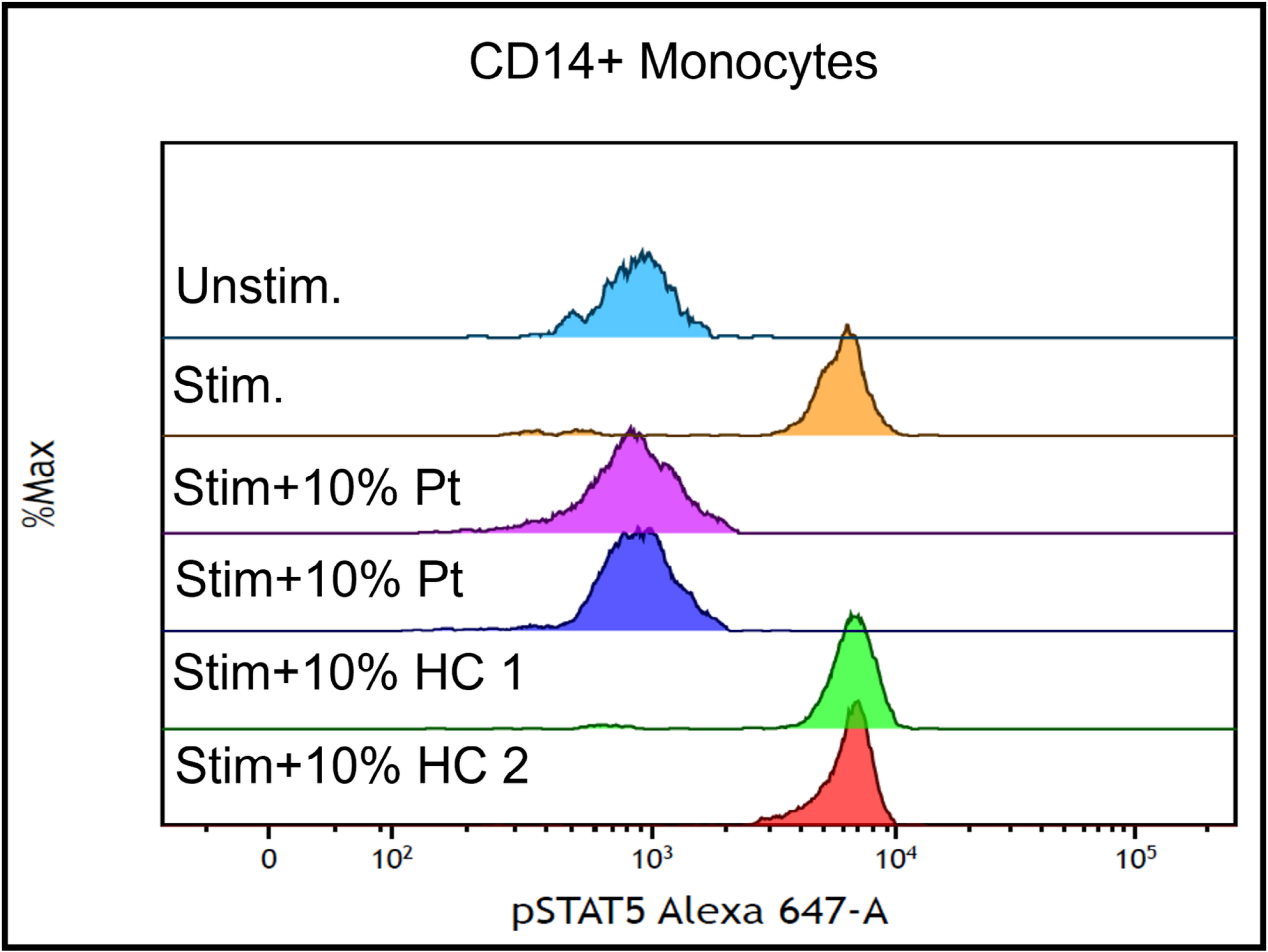
Flow cytometry histogram plot of CD14+ monocytes with either unstimulated (unstim), stimulated (stim) with GM‐CSF 10 ng/mL, stained with phosphoSTAT5, in the presence of either patient serum (Pt, replicates) or healthy control (HC) serum (single replicate of two separate subjects), demonstrating complete neutralisation of GM‐CSF by patient serum.

The patient re‐presented 4‐weeks later with severe hypoxic respiratory failure. Investigation for infection (respiratory viral panel, sputum culture and blood culture) and immunodeficiency (lymphocyte subsets, immunoglobulin levels, HIV‐serology and HTLV‐serology) were negative. In the outpatient period, the patient reported continued cigarette and cannabis smoking and had not returned to work nor had further exposure to native flora. A repeat CT chest demonstrated progressive ground glass opacities with inter‐lobular septal thickening, the classical crazy‐paving pattern (Figure 1C). Repeat bronchoscopy was performed and returned cedar‐coloured fluid, however cultures and CMV titres were unremarkable. Despite broad spectrum anti‐microbials and corticosteroids, respiratory status deteriorated requiring a fraction of inspired oxygen (FiO_2_) of 80% via high‐flow nasal cannula (HFNC). The rare association between cryptococcosis and PAP due to GM‐CSF antibodies was considered. Repeat bronchoscopy with lavage of cedar‐brown fluid contained PAS‐positive sediment using a PAS with diastase protocol. Testing for anti‐GM‐CSF antibodies was performed by the local immunopathology laboratory using a neutralisation assay. Briefly, healthy donor peripheral mononuclear cells were incubated with recombinant human GM‐CSF in the presence of patient or healthy donor serum. Expression of pSTAT5 relative to positive and negative controls was measured to determine the presence of GM‐CSF antibodies, confirming the diagnosis of autoimmune‐PAP (Figure 2).

**FIGURE 3:**
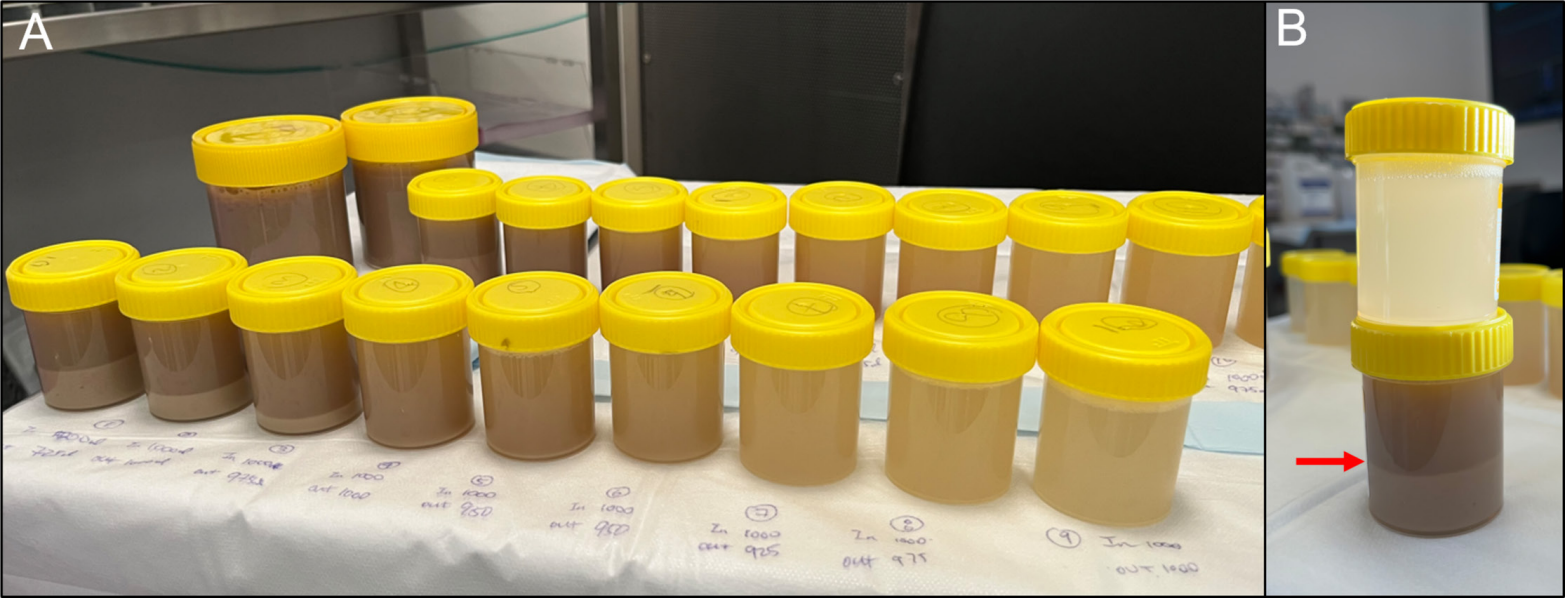
Whole lung lavage fluid specimens. Jug filled via efferent limb and aliquoted into pots with excess fluid discarded. This allows visual estimation of sediment volume and analysis of colour and clarity of lavage fluid. (A) sequential lavage samples from left lung (front) and right lung (back) from 1st litre (left) to final litre (right). (B) juxtaposes initial fluid sample (bottom) with final fluid sample (top). Red arrow indicates sediment: Fluid interface.

**FIGURE 4:**
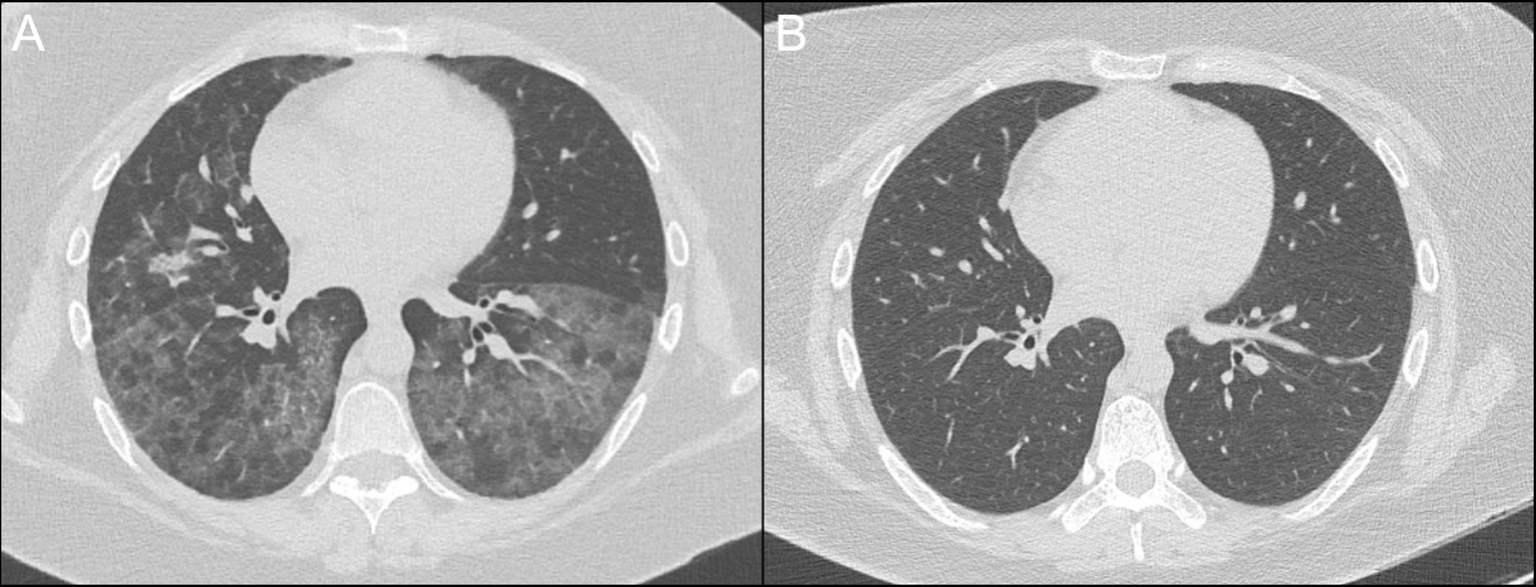
CT chest axial slices. (A) 8‐weeks post‐bilateral whole lung lavage demonstrating recurrence of bilateral ground glass opacities. (B) 5‐months post‐right lung lavage with sustained radiographic resolution of ground glass changes bilaterally.

Cardiac anaesthetics, thoracic medicine and intensive care specialists made consensus decision to perform bilateral WLL on VV‐ECMO due to severity of respiratory failure and rapid progression. A dual‐lumen endotracheal tube was inserted to left main bronchus and position confirmed by bronchoscopy. Right lung was selected for lavage first due to the greater degree of infiltrate seen on CT chest (Figure 1C). 1‐L aliquots of warmed normal saline were instilled from a 50 cm height above the thorax via passive instillation and both afferent and efferent limbs were clamped. With each litre of lavage in situ, chest percussion was performed by chest physiotherapists and patient position was manipulated using electronic bed to optimise return from different subsegments. The efferent limb was unclamped, and drainage volume, colour and sediment were recorded (Figure 3). This process was repeated for the left lung until no sediment was seen and effluent transparent. A total of 14,080 mL instilled into the right lung with 13,125 mL return. 9000 mL instilled into the left lung with 8500 mL return. Airways to third generation bronchi were cleared via flexible bronchoscopy. The patient had rapid response to WLL, transitioning from HFNC to maintaining SpO_2_ 95% on an FiO_2_ of 21% post‐ECMO decannulation (Corresponding CT—Figure 1D). Left lung lavage was performed 3 months later due to recurrent radiographic ground glass opacities (Figure 4A) and symptomatic dyspnoea. PAP has since remitted on follow up CT chest after a further 5 months (Figure 4B).

## 3 Discussion

Pulmonary alveolar proteinosis is a rare disease with a global prevalence estimated to be seven cases per million [1]. Autoimmune‐PAP is characterised by the development of GM‐CSF antibodies that impair macrophage function leading to the accumulation of proteinaceous material in the alveolar space, neutrophil dysfunction, innate immune deficiency, and secondary infections with atypical organisms [1]. The identification of PAP can be a diagnostic challenge due to the non‐specific radiographic changes that are seen early in the disease prior to the development of the characteristic crazy‐paving pattern. The insidious presentation, which is often clinically superseded by the emergent infection further exacerbates the diagnostic uncertainty. In our case, the diagnosis was complicated by the close temporal relationship between the cryptococcus infection and the development of PAP such that the cryptococcus was still undergoing active management at the time of diagnosis. The development of immune reconstitution in addition to other distracting sequelae of CMV pneumonitis and contaminating airway organisms on lavage cultures enhanced the diagnostic challenge. A high index of suspicion is needed for those presenting with atypical infections, even in the absence of typical radiographic findings. As *C. gattii* is endemic in Australia, a greater degree of scrutiny is required in the Australian population. It is important to acknowledge that organisms other than *Cryptococcus* spp. are more commonly associated with the development of PAP. A comprehensive literature review spanning 1950–2010 by Punatar et al., including 75 cases of PAP and infection, indicated that Nocardia spp. accounted for 43% of preceding infections, mycobacteria 37% and fungal infections accounted for 20% of which *Cryptococcus* spp. only represented 33% [10]. There is a paucity of literature demonstrating the proportion of patients who present with a concurrent diagnosis of PAP and cryptococcal infection, however, there are rare reports of cryptococcal infection in those with established PAP dating back several decades [11, 12, 13, 14].

A review of the English literature between 1950 and March 2024 using PubMed database focusing on PAP associated with cryptococcus was conducted using the search terms ‘cryptococcosis or cryptococcus or cryptococcal’, ‘pulmonary alveolar proteinosis’ or ‘PAP’ and ‘GM‐CSF antibodies or granulocyte‐macrophage colony‐stimulating factor antibodies’. References cited by articles were also analysed. The demographic data, presenting symptoms, site of infection, diagnostic investigations, time to diagnosis and management are summarised in Table 1. Eight cases were identified, including our case, where diagnosis of PAP occurred distinctly after the initial presentation of cryptococcosis. The distribution of cases was international and in locations where *C. gattii* is endemic, including North America, South America, Western Europe, and Australasia. Most patients presented with symptoms of CNS disease and were found to have a dense pulmonary consolidation with or without cavitation on CT chest. The median time to diagnosis of PAP following cryptococcosis was 36‐months, typically with widespread ground glass change or crazy‐paving pattern, bronchoalveolar lavage with PAS‐positive sediment and elevated levels of GM‐CSF‐antibodies. In our case, diagnosis occurred within 6‐weeks and progressed quickly to severe hypoxic respiratory failure. We hypothesise that this may be attributable to repeated airway insults, such as the continued tobacco smoking, which led to increased accumulation of sediment within the alveolar space as evidenced by the reported association of smoking with increased PAP severity as well as the direct effect of cigarette smoke on macrophage functions [15, 16]. Cannabis has also been associated with impaired pulmonary immune function including macrophages, as summarised by Preteroti et al. [17]. There were no other attributable causes for the rapid progression identified on review of the daily routine or treatment, which consisted of oral fluconazole, valganciclovir, 40 mg prednisolone, pantoprazole, and trimethoprim‐sulfamethoxazole. Interestingly, the opaque dark‐brown colour of the lavage fluid differs from the classic milky fluid and may have corresponded with the presence of airway contamination by smoking of tobacco and cannabis products. It is important to avoid discounting diagnoses by the atypical appearance of lavage fluid in these cases. The sustained clinical and radiologic remission of PAP 5‐months after right lung lavage corresponds with the cessation of cigarette and cannabis smoking and may reflect slowing of disease progression or possible resolution, which can be seen in up to 5%–7% of cases [1].

**TABLE 1:** Literature review of patients presenting with autoimmune PAP following cryptococcal infection [3, 4, 5, 6, 7, 8, 9].

Case	Age	Sex	Clinical presentation	Background	Site	Organism	Initial CT chest	Diagnostic CT chest	BAL	GM‐CSF Ab	Biopsy modality	Time‐to‐diagnosis	Management
1	50	M	Headache, fever	Nil	CNS, Pulmonary	*C. gattii*	R upper lobe hilar mass	Crazy paving	PAS−	—	Wedge biopsy	15 months	Monitoring
2	42	M	Headache, fever, facial paralysis	Active smoker (20 pack‐year)	CNS, Pulmonary	*C. gattii*	R hilar mass	Crazy paving	PAS+	Yes	Transthoracic needle biopsy	36+ months	Monitoring
3	44	M	—	Reformed smoker	CNS, Pulmonary	*C. gattii*	L apical mass with peri‐bronchial ground glass	Crazy paving	PAS+	Yes	Transbronchial cryobiopsy	72+ months	Bilateral WLL
4	20	F	Headache, fever, diplopia, vomiting, confusion	Nil	CNS, Pulmonary	*C. neoformans*	L lower lobe mass	Ground glass change	PAS+	Yes	Transbronchial forceps biopsy	24+ months	Bilateral WLL
5	47	M	Cough	Nil	Pulmonary	*C. neoformans*	Perihilar mass	Ground glass change	PAS+	Yes	—	40+ months	Monitoring
6	44	M	Headache	Nil	CNS, Pulmonary	*C. gattii*	L lower lobe cavitating mass	Crazy paving	PAS+	Yes	Transbronchial forceps biopsy	12 months	Corticosteroids, monitoring
7	48	M	Haemoptysis	Nasal polyposis	CNS, Pulmonary	*C. gattii*	R upper lobe cavitating mass	Ground glass change	—	Yes	—	48+ months	Monitoring
Current	24	F	Seizure, headache	Active smoker (5 pack‐year)	CNS, Pulmonary	*C. gattii*	R lower lobe consolidation	Crazy paving	PAS+	Yes	—	1.5 months	Bilateral WLL+R single lung lavage

Abbreviations: BAL: bronchoalveolar lavage; CNS: central nervous system; F: female; L: left; M: male; PAS: periodic‐acid Schiff; R: right; WLL: whole lung lavage.

Due to the rapid progression of severe respiratory failure and the delay to diagnosis of PAP in this case, an opportunity to perform single WLL in a controlled setting was lost. Thus, a decision was made to perform bilateral WLL on VV‐ECMO. The case demonstrates the utility of bilateral WLL even in the most severe cases of PAP. This approach has been rarely reported in the literature with similarly positive outcomes [18, 19, 20, 21]. Given our patient's severe and complex condition, a multidisciplinary approach was crucial to determine the best treatment course amid diagnostic uncertainty. The risks of bilateral whole lung lavage were acknowledged prior to procedure and protocol devised from the literature given a shortage of local expertise. The decision to passively instil fluid from a height of 50 cm above patient aimed to reduce the risk of barotrauma and careful monitoring of input and output volumes is essential to minimise rates of pneumothorax.

In conclusion, we present the case of an Aboriginal Australian woman who was diagnosed with autoimmune‐PAP after presenting with *C. gattii* meningitis. This case represents a rare disease with rapid clinical course. The short interval between cryptococcal infection and development of clinical PAP is unique and the diagnostic challenge of this disease highlights the need for multi‐disciplinary, collaborative approaches to diagnosis and management. It remains unclear whether *C. gattii* could contribute to the development of PAP, or if subclinical PAP is present at the time of infection. WLL on VV‐ECMO has been infrequently documented in the literature and can lead to rapid remission of disease. The constellation of preceding infection with a classic microorganism, presence of ground glass change or crazy paving pattern and opaque bronchoalveolar lavage fluid should prompt physicians to include this diagnosis in the differential. Clinicians should be aware of the potential wide temporal dispersion between cryptococcal infection and radiographic development of PAP.

## Author Contributions

**Samuel Cartmel Brookes:** collection of clinical data, drafted manuscript and performed literature review. **Alexandra Sadler:** collection of clinical data and assisted drafting manuscript. **Alexander Troelnikov:** contributed from immunology perspective, provided diagnostic input and assisted in writing methodology of anti‐GM‐CSF antibody testing and figure. **Pravin Hissaria:** reviewed manuscript, provided diagnostic input and contributed to immunology perspective. **Michael Vickery Brown:** extensively reviewed manuscript, assisted with literature review and contributed to whole lung lavage. **Phan Nguyen:** reviewed manuscript and interventional pulmonology perspective about whole lung lavage. **Julia Kim:** reviewed manuscript and assisted with nursing perspective of whole lung lavage. **Paroma Sarkar:** extensively reviewed manuscript, assisted with literature review and provided input from a general respiratory and respiratory infectious disease perspective.

## Ethics Statement

The authors declare that appropriate written informed consent was obtained for the publication of this manuscript and accompanying images.

## Conflicts of Interest

Phan Nguyen is an Editorial Board member of Respirology Case Reports and a co‐author of this article. He was excluded from all editorial decision‐making related to the acceptance of this article for publication.

## Data Availability

The data that support the findings of this study are available from the corresponding author upon reasonable request.

## References

[1] C. McCarthy , B. C. Carey , and B. C. Trapnell , “Autoimmune Pulmonary Alveolar Proteinosis,” American Journal of Respiratory and Critical Care Medicine 205 (2022): 1016–1035, 10.1164/rccm.202112-2742SO. 35227171 PMC9851473

[2] K. J. Kwon‐Chung , J. A. Fraser , T. L. Doering , et al., “ *Cryptococcus neoformans* and *Cryptococcus gattii*, the Etiologic Agents of Cryptococcosis,” Cold Spring Harbor Perspectives in Medicine 4, no. 7 (2014): a019760, 10.1101/cshperspect.a019760. 24985132 PMC4066639

[3] J. Quah , T. B. Low , and R. Fong , “Disseminated *Cryptococcus gattii* Infection Preceding Onset of Pulmonary Alveolar Proteinosis,” Respirology Case Reports 6, no. 7 (2018): e00357, 10.1002/rcr2.357. 30083345 PMC6071436

[4] S. Demir , N. Chebib , F. Thivolet‐Bejui , and V. Cottin , “Pulmonary Alveolar Proteinosis Following Cryptococcal Meningitis: A Possible Cause?,” BML Case Reports 2018 (2018), 10.1136/bcr-2017-222940. PMC5878345 29592982

[5] W. T. Lim , S. M. T. Priyangika , and J. S. K. P. Karunarthne , “Crazy‐Paving Pattern: A Rare Case of Autoimmune Pulmonary Alveolar Proteinosis (PAP) With Positive Anti‐GM‐CSF Antibody Following Cryptococcal Infection in an Otherwise Healthy Individual and Review of Literature,” European Journal of Respiratory Medicine 3, no. 2 (2021): 200–205, 10.31488/EJRM.116.

[6] L. B. Rosen , A. F. Freeman , L. M. Yang , et al., “Anti‐GM‐CSF Autoantibodies in Patients With Cryptococcal Meningitis,” Journal of Immunology 190 (2013): 3959–3966, 10.4049/jimmunol.1202526. PMC3675663 23509356

[7] E. Lee , C. Miller , A. Ataya , and T. Wang , “Opportunistic Infection Associated With Elevated GM‐CSF Autoantibodies: A Case Series and Review of the Literature,” Open Forum Infectious Diseases 9, no. 5 (2022), 10.1093/ofid/ofac146. PMC9070348 35531378

[8] B. Stevenson , C. Bundell , S. Mulrennan , A. McLean‐Tooke , R. Murray , and A. Brusch , “The Significance of Anti‐Granulocyte‐Macrophage Colony‐Stimulating Factor Antibodies in Cryptococcal Infection: Case Series and Review of Antibody Testing,” Internal Medicine Journal 49 (2019): 1446–1450, 10.1111/imj.14637. 31713345

[9] S. Y. Wang , Y. F. Lo , H. P. Shih , et al., “ *Cryptococcus gattii* Infection as the Major Clinical Manifestation in Patients With Autoantibodies Against Granulocyte–Macrophage Colony‐Stimulating Factor,” Journal of Clinical Immunology 42, no. 8 (2022): 1730–1741, 10.1007/s10875-022-01341-2. 35947322

[10] A. D. Punatar , S. Kusne , J. E. Blair , M. T. Seville , and H. R. Vikram , “Opportunistic Infections in Patients With Pulmonary Alveolar Proteinosis,” Journal of Infection 65 (2012): 173–179, 10.1016/j.jinf.2012.03.020. 22484272

[11] F. Bergman and F. Linell , “Cryptococcosis as a Cause of Pulmonary Alveolar Proteinosis,” Acta Pathologica et Microbiologica Scandinavica 53 (1961): 217–224, 10.1111/j.1699-0463.1961.tb00403.x. 13867733

[12] S. H. Rosen , B. Castleman , A. A. Liebow , F. M. Enzinger , and R. T. N. Hunt , “Pulmonary Alveolar Proteinosis,” New England Journal of Medicine 258 (1958): 1123–1142, 10.1056/NEJM195806052582301. 13552931

[13] W. A. Sunderland , R. A. Campbell , and M. J. Edwards , “Pulmonary Alveolar Proteinosis and Pulmonary Cryptococcosis in an Adolescent Boy,” Journal of Pediatrics 80 (1972): 450–456, 10.1016/s0022-3476(72)80503-1. 5060458

[14] M. G. Lee , H. Spencer , W. F. Clarke , B. N. Rao , M. Lowe , and M. Nelson , “Pulmonary Alveolar Proteinosis in Jamaica,” West Indian Medical Journal 31, no. 3 (1982): 103–110. 7179933

[15] S. T. Lugg , A. Scott , D. Parekh , B. Naidu , and D. R. Thickett , “Cigarette Smoke Exposure and Alveolar Macrophages: Mechanisms for Lung Disease,” Thorax 77 (2022): 94–101, 10.1136/thoraxjnl-2020-216296. 33986144 PMC8685655

[16] J. A. Hwang , J. H. Song , J. H. Kim , et al., “Clinical Significance of Cigarette Smoking and Dust Exposure in Pulmonary Alveolar Proteinosis: A Korean National Survey,” BMC Pulmonary Medicine 17 (2017): 147, 10.1186/s12890-017-0493-4. 29162083 PMC5697136

[17] M. Preteroti , E. T. Wilson , D. H. Eidelman , and C. J. Baglole , “Modulation of Pulmonary Immune Function by Inhaled Cannabis Products and Consequences for Lung Disease,” Respiratory Research 24 (2023): 95, 10.1186/s12931-023-02399-1. 36978106 PMC10043545

[18] A. D. L. Sihoe , V. M. W. Ng , R. W. T. Liu , and L. C. Cheng , “Pulmonary Alveolar Proteinosis in Extremis: The Case for Aggressive Whole Lung Lavage With Extracorporeal Membrane Oxygenation Support,” Heart, Lung & Circulation 17 (2008): 69–72, 10.1016/j.hlc.2006.11.007. 17337244

[19] K. H. Kim , J. H. Kim , and Y. W. Kim , “Use of Extracorporeal Membrane Oxygenation (ECMO) During Whole Lung Lavage in Pulmonary Alveolar Proteinosis Associated With Lung Cancer,” European Journal of Cardio‐Thoracic Surgery 26 (2004): 1050–1051, 10.1016/j.ejcts.2004.08.005. 15519209

[20] N. Hasan , S. Bagga , J. Monteagudo , et al., “Extracorporeal Membrane Oxygenation to Support Whole‐Lung Lavage in Pulmonary Alveolar Proteinosis: Salvage of the Drowned Lungs,” Journal of Bronchology & Interventional Pulmonology 20, no. 1 (2013): 41–44, 10.1097/LBR.0b013e31827ccdb5. 23328142

[21] S. Chauhan , K. P. Sharma , A. K. Bisoi , R. Pangeni , K. Madan , and Y. S. Chauhan , “Management of Pulmonary Alveolar Proteinosis With Whole Lung Lavage Using Extracorporeal Membrane Oxygenation Support in a Postrenal Transplant Patient With Graft Failure,” Annals of Cardiac Anaesthesia 19 (2016): 379, 10.4103/0971-9784.179627. 27052091 PMC4900367

